# Supplementary material for: Bioaccessibility and Intestinal Transport of Tebuconazole in Table Grape by Using In Vitro Digestion Models
Source: Foods. 2022 Dec 5;11(23):3926. doi: 10.3390/foods11233926 (PMC9740649; doi:10.3390/foods11233926)
Supplement: Supplementary file 1 [file foods-11-03926-s001.zip › foods-1974468-supplementary.pdf]

Table S1. Reagents, pH value, solid-liquid ratio and digestion time of different in vitro simulated digestion methods.

Simulation method	Simulated digestive fluid	Reagent（The content in 1L water）	PH value	Solid-Liquid ratio（%）	Digestion time（h）
DIN	Gastric juice	1 g Pepsin, 3 g Mucin, 2.9 g NaCl, 0.7 g KCl, 0.27 gKH_2PO_4	2	1:10	2
	Intestinal juice	9 g Bile powder, 9 g Pancreatin, 0.3 g Trypsin,0.3 g Urea,0.3 g NaCl, 0.3 g KCl, 0.5 g $CaCl_2$, 0.2 g $MgCl_2$	7.5	1:20	6
IVG	Gastric juice	10 g Pepsin, 8.77 g NaCl	1.8	1:10	1
	Intestinal juice	3.5 g Bile powder, 0.35 g Trypsin	5.5	1:20	1
PBET	Gastric juice	1.25 g Pepsin, 0.5 g NaCl, 0.5 g Sodium citrate, 0.42 mL Lactic acid, 0.5 mL acetic acid	2.5	1:10	1
	Intestinal juice	1.75 g Bile powder, 0.5 g Pancreatin	7.5	1:20	4
SBRC	Gastric juice	30.03 g Glycine	1.5	1:10	1
	Intestinal juice	1.75 g Bile powder, 0.5 g Pancreatin	7	1:20	4
SHIME	Nutrient solution	1 g Peptone, 1 gD-(+)-Galactose, 1 g Xylan, 2 g Pectin, 3 g Soluble starch, 4 g Mucin, 0.5 g L-cysteine, 0.4 g Glucose, 3 g Yeast Extract		-	
	Gastric acid	0.089 g of pepsin is dissolved in 1 L of 1 mol/L hydrochloric acid solvent			
	Gastric juice	Add 125 mL of gastric acid to 1 L of nutrient solution	2.5	1:40	2
	Intestinal juice	12.5 g $NaHCO_3$, 6 g Bile powder, 0.9 g Pancreatin are added to 1 L nutrient solution	6.5	1:40	2

NaCl: Sodium chloride; KCl: Potassium Chloride; $MgCl_2$: Magnesium chloride; $CaCl_2$: Calcium chloride; KH_2PO_4: Potassium dihydrogen phosphate; HCl: Hydrochloric acid; $NaHCO_3$: Sodium bicarbonate.

Table S2. Linear range, calibration curves,correlation coefficients (R^2) and Limits of quantificiation(LOQs) of tebuconazole in grapes and simulated digestive gastrointestinal juice.

Matrix	Linear range (mg/kg)	Linear equation	Correlation coefficients(R^2)	LOQs	Recovery rate(%) (RSDs) (n=5)		
					0.02mg/kg	0.2 mg/kg	2 mg/kg
Grape	0.005-1	Y=14938.4X+7140.32	0.998706	0.005	71.10 (3.04)	78.73 (5.91)	72.44 (4.80)
DIN-G	0.01-1	Y=1054.48X+4193.51	0.991032	0.01	73.50 (4.19)	104.19 (5.17)	89.40 (2.61)
DIN-I	0.05-1	Y=562.945X-62.3157	0.999671	0.05	105.20 (3.25)	102.01 (3.76)	94.07 (5.18)
IVG-G	0.05-2	Y=1382.86X+2253.5	0.99967	0.05	98.60 (4.99)	100.97 (3.70)	87.06 (3.58)
IVG-I	0.05-2	Y=551.282X+7503.98	0.998532	0.05	77.00 (4.19)	92.73 (11.38)	88.84 (3.60)
PBET-G	0.01-1	Y=1006.55X+4667.17	0.999074	0.01	116.80 (3.35)	103.91 (2.84)	110.35 (2.79)
PBET-I	0.005-1	Y=672.693X+876.815	0.998914	0.005	113.80 (5.90)	116.33 (3.88)	89.29 (1.70)
SBRC-G	0.02-1	Y=1302.21X+5630.56	0.999511	0.02	88.70 (5.95)	98.48 (6.91)	76.96 (4.86)
SBRC-I	0.01-2	Y=1303.96X+6157.78	0.999033	0.01	80.40 (5.71)	114.33 (1.84)	101.23(2.74)
SHIME-G	0.05-1	Y=1467.75X+1651.27	0.999421	0.05	115.50 (4.72)	113.13 (1.40)	108.91 (1.47)
SHIME-I	0.002-2	Y=673.679X-604.659	0.996138	0.002	81.90 (2.18)	104.76 (2.37)	99.17 (0.79)

G: Gastic phase; I: Intestinal phase; RSDs: Relative Standard Deviation.
